# Comparative genomic analysis of *Vibrio parahaemolyticus*: serotype conversion and virulence

**DOI:** 10.1186/1471-2164-12-294

**Published:** 2011-06-06

**Authors:** Yuansha Chen, O Colin Stine, Jonathan H Badger, Ana I Gil, G Balakrish Nair, Mitsuaki Nishibuchi, Derrick E Fouts

**Affiliations:** 1 Emerging Pathogens Institute, University of Florida, Gainesville, FL, USA; 2 Department of Pathology, University of Florida, Gainesville, FL, USA; 3 School of Medicine, University of Maryland, Baltimore, MD, USA; 4 Instituto de Investigación Nutricional, Peru; 5 National Institute of Cholera and Enteric Diseases, Kolkata, India; 6 Kyoto University, Kyoto, Japan; 7 The J. Craig Venter Institute, Rockville, MD, USA

## Abstract

**Background:**

*Vibrio parahaemolyticus *is a common cause of foodborne disease. Beginning in 1996, a more virulent strain having serotype O3:K6 caused major outbreaks in India and other parts of the world, resulting in the emergence of a pandemic. Other serovariants of this strain emerged during its dissemination and together with the original O3:K6 were termed strains of the pandemic clone. Two genomes, one of this virulent strain and one pre-pandemic strain have been sequenced. We sequenced four additional genomes of *V. parahaemolyticus *in this study that were isolated from different geographical regions and time points. Comparative genomic analyses of six strains of *V. parahaemolyticus *isolated from Asia and Peru were performed in order to advance knowledge concerning the evolution of *V. parahaemolyticus*; specifically, the genetic changes contributing to serotype conversion and virulence. Two pre-pandemic strains and three pandemic strains, isolated from different geographical regions, were serotype O3:K6 and either toxin profiles (*tdh+*, *trh*-) or (*tdh-*, *trh*+). The sixth pandemic strain sequenced in this study was serotype O4:K68.

**Results:**

Genomic analyses revealed that the *trh*+ and *tdh*+ strains had different types of pathogenicity islands and mobile elements as well as major structural differences between the *tdh *pathogenicity islands of the pre-pandemic and pandemic strains. In addition, the results of single nucleotide polymorphism (SNP) analysis showed that 94% of the SNPs between O3:K6 and O4:K68 pandemic isolates were within a 141 kb region surrounding the O- and K-antigen-encoding gene clusters. The "core" genes of *V. parahaemolyticus *were also compared to those of *V. cholerae *and *V. vulnificus*, in order to delineate differences between these three pathogenic species. Approximately one-half (49-59%) of each species' core genes were conserved in all three species, and 14-24% of the core genes were species-specific and in different functional categories.

**Conclusions:**

Our data support the idea that the pandemic strains are closely related and that recent South American outbreaks of foodborne disease caused by *V. parahaemolyticus *are closely linked to outbreaks in India. Serotype conversion from O3:K6 to O4:K68 was likely due to a recombination event involving a region much larger than the O-antigen- and K-antigen-encoding gene clusters. Major differences between pathogenicity islands and mobile elements are also likely driving the evolution of *V. parahaemolyticus*. In addition, our analyses categorized genes that may be useful in differentiating pathogenic Vibrios at the species level.

## Background

*Vibrio parahaemolyticus *is a halophilic bacterium which has long been recognized [1] as a human pathogen that causes gastroenteritis and, occasionally, wound infections and sepsis in immunocompromised patients. It is the leading etiologic agent for bacterial foodborne disease in Japan and other parts of Asia, and it is the most common bacterial cause of seafood-associated disease in the United States. Prior to 1996, there was no specific serotype of *V. parahaemolyticus *that was associated with disease outbreaks, and the bacterium had never been reported to cause a pandemic. However, during that year, a major outbreak occurred in India, > 50% of the *V. parahaemolyticus *strains isolated from patients were serotype O3:K6 [2]. Also, the outbreak rapidly spread to other countries in Asia, South America, North America, Africa and Europe, resulting in a pandemic affecting tens of thousands of people [2,3]. During its global dissemination, > 20 serovariants (including O3:K6, O4:K68, O1:K25, O1:KUT [untypable], and others [2,4,5] rapidly evolved from the original pandemic O3:K6 strain. The pandemic O3:K6 and its serovariants are termed strains of the pandemic clone.

A thermostable direct hemolysin (TDH) is recognized [6] as the most important virulence factor of *V. parahaemolyticus*, and a TDH-related hemolysin (TRH) is believed to account for the virulence of strains that do not produce TDH. Prior whole-genome sequencing [7,8] of a serotype O3:K6, pandemic isolate designated RIMD2210633 identified two type III secretion systems (T3SS). T3SSI is present in all *V. parahaemolyticus *isolates examined and is required for the bacterium's cytolytic activity [8]; whereas, T3SSII is required for enterotoxicity and is located in the *tdh*-containing pathogenicity island [7,8].

Outbreaks of diarrheal disease caused by *V. parahaemolyticus *may pose a significant health threat. Thus far, the most affected country (other than India) has been Chile, where > 10,000 cases were reported during 2005. This observation suggests that, under appropriate conditions, *V. parahaemolyticus *may cause large-scale outbreaks comparable to those elicited by *V. cholerae*. At the present time, the reasons for the pandemic strains' rapid increase in virulence/prevalence have not been rigorously determined. In addition, the mechanism(s) for rapid serotype conversion warrant further study. Furthermore, it is not clear whether the virulence mechanisms of *tdh*+ and *trh*+ strains are similar. Therefore, in order to address some of these questions, we performed rigorous genomic analyses of two pre-pandemic and four pandemic isolates of *V. parahaemolyticus*.

## Results and Discussion

### Comparative genomics of *V. parahaemolyticus*

Prior to this study, an O3:K6 pandemic isolate (strain RIMD2210633) was sequenced to completion [7] and an O3:K6 non-pandemic isolate (strain AQ3810) was sequenced to draft status [9]. In this study, we sequenced four additional isolates of *V. parahaemolyticus *to at least 8-fold draft coverage, for a total of six clinical isolates; two non-pandemic and four pandemic (Table 1). The two non-pandemic strains, AQ3810 and AQ4037, were isolated in 1983 and 1985, respectively, and both originated from Southeast Asia. Throughout the remainder of this study, we will refer to these two non-pandemic isolates as "pre-pandemic" because they were isolated prior to the documented start of the pandemic. Three of the pandemic isolates were from Southeast Asia, including strain RIMD2210633 in 1996, strain AN5034 in 1998, and strain K5030 in 2005, while the fourth pandemic isolate (strain Peru466) was isolated from Peru in 1996. Therefore, the isolates represented two geographic areas where major outbreaks occurred. In addition, they also have different serotypes and toxin profiles. All of the pandemic strains were (*tdh*+ *trh*-) and the pre-pandemic strains were either (*tdh*+ *trh*-) or (*tdh*- *trh*+), thus representing two potentially different virulence mechanisms. To improve our understanding of the pandemic clone's evolution during their global dissemination, the genome of a Peruvian isolate (strain Peru466) [10] was sequenced and compared to the genomes of Asian isolates collected at different time points during the pandemic. In the later stage of the pandemic, there were fewer cases of infection in South Asia; thus, *V. parahaemolyticus *isolated during this time seems to be less virulent (Nair, personal observation). Therefore, an isolate (strain K5030) collected in 2005 from India was included and considered a "less virulent" late stage pandemic isolate in this study. Also, the genome of a never-before-sequenced serotype O4:K68 pandemic isolate (strain AN5034) was characterized in order to advance our understanding of the mechanism for its serotype conversion.

**Table 1:** Six *V. parahaemolyticus *strains analyzed during this study

Year	Strain	Source	Serotype	*tdh*	*trh*	# contigs	Contig N50^†§ ^(bp)	Max. Contig	Reference
1983^‡^	AQ3810	Singapore	O3:K6	+	-	1037	52609	295134	[9]
1985^‡^	AQ4037	Maldives	O3:K6	-	+	164	67710	241746	This study
1996	RIMD2210633	Thailand	O3:K6	+	-	2	N.A.	3288558	[7]
1996	Peru466	Peru	O3:K6	+	-	149	81497	273858	This study
1998	AN5034	Bangladesh	O4:K68	+	-	54	346246	1183081	This study
2005	K5030	India	O3:K6	+	-	164	62978	657114	This study

^†^The contig N50 is the length of the smallest contig in the set that contains the fewest (largest) contigs whose combined length represents at least 50% of the assembly [41].

^§^The contig N50 was calculated from the Celera assembly, not the contigs submitted to Genbank.

^‡^Pre-pandemic years.

N.A. Not Applicable.

**Figure 1:**
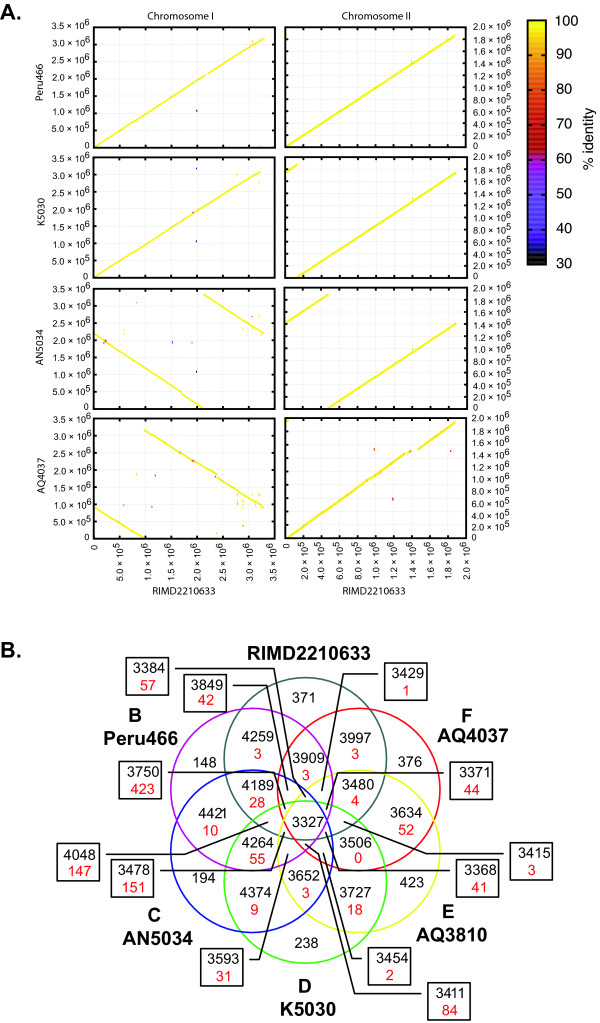
**Whole-genome comparison of six *V. parahaemolyticus *strains**. Panel A: Colored lines denote the percent identities of protein translations, and they are plotted according to their locations in the reference strain (RIMD2210633) and query strain's genomes. Panel B: Venn diagrams indicate the number of shared proteins (black) and unique proteins (red) within a particular relationship for all six *V. parahaemolyticus *strains.

The pan genome of the six *V. parahaemolyticus *strains we examined had 6,616 chromosomal coding genes, and each individual genome (excluding plasmids) had an average of 4,673 coding genes (Figure 1). Three thousand twenty eight genes, ca. 71% of the coding genes were present in all the strains (Additional file 1). However, that number may be lower than the actual number because the genomes, except for the genome of RIMD2210633, were not sequenced to completion. Therefore, some of the open-reading frames (ORFs) that bordered contigs may have failed to meet the cut-offs and, subsequently, were treated as not present. The four newly sequenced genomes displayed a high degree of synteny with RIMD2210633 (Figure 1). There was very little rearrangement of the genome of the pre-pandemic strain AQ4037 and essentially no rearrangement in the pandemic strains. Because the gaps are not closed for five of the genomes, our report of synteny represents our best estimation.

### Super integron

*V. parahaemolyticus *harbors a super integron (SI) on chromosome I. The SI is about 48 kb long and contains ca. 77 ORFs, which is much smaller than the SIs in *V. cholerae *(120 kb) and in *V. vulnificus *(138 kb). Most of the ORFs in the SI regions encode hypothetical proteins. The SI integrases were identical in the six *V. parahaemolyticus *strains examined, but the cassettes in the SI regions of the pre-pandemic strains varied greatly from those of the pandemic strains. For example, only 24 and 28 of the 77 ORFs in the pandemic strains' SI regions were present in those of pre-pandemic strains AQ3810 and AQ4037, respectively. However, the cassettes in the SI regions of the four pandemic strains were nearly identical; i.e., they contained only a few point mutations. The only exception was isolate K5030, which had an additional six hypothetical proteins inserted between the integrase and the rest of the cassettes. These observations indicate that the integrase is active in *V. parahaemolyticus *and contributes to species evolution. However, the fact that its SI region is smaller than those of other pathogenic *Vibrio *species, and the presence of highly conserved cassettes in the pandemic strains, suggests that the genomes of *V. parahaemolyticus *may be more stable than those of other pathogenic Vibrios.

### Pathogenicity islands, prophages, and integrated elements

**Figure 2:**
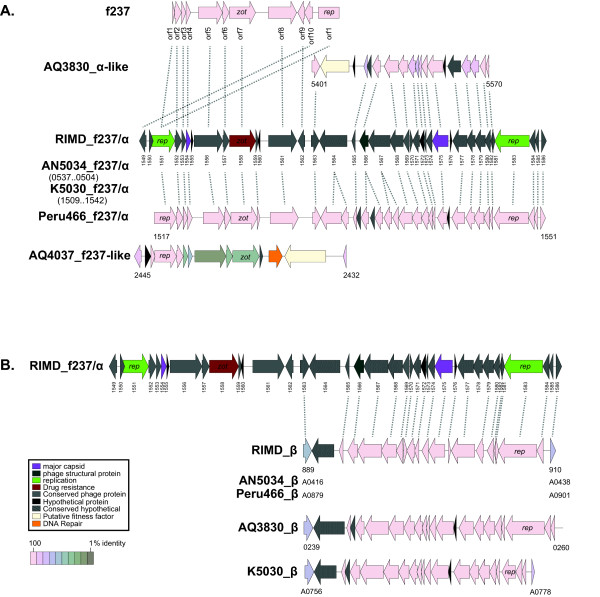
**Linear illustration of f237-like prophage and juxtaposed regions**. Depicted are linear representations of ORFs found on chromosome I of each query genome with similarity to the f237-like prophage in RIMD2210633 (A). Those regions found on chromosome II of query genomes with similarity only to the alpha region of RIMD_f237/α are also shown (B). Query ORFs are colored by protein percent identity to RIMD2210633 proteins (see key). The reference RIMD_f237/α ORFs and query ORFs with no match to RIMD_f237/α ORFs are colored by function role categories as noted in the boxed key.

Only the pandemic strains examined in this study contained the pathogenicity islands previously described [7] for *V. parahaemolyticus *(Table 2). In addition, we detected various prophages and integrated elements using Phage_Finder [11]. Prophage f237, which has been widely used as a genetic marker for the pandemic clone [12], was present in chromosome I (loci VP1549-1562 in strain RIMD2210633) of all the pandemic strains we examined, but it was absent from the pre-pandemic strains (Figure 2A). However, a prophage similar to f237 was present in pre-pandemic strain AQ4037, in the same location occupied by f237 in the pandemic strains (Figure 2A). In addition to f237, another prophage was identified adjacent to f237 (loci VP1563-1586 in strain RIMD2210633) in all of the pandemic strains and in pre-pandemic strain AQ3810, but it was absent from strain AQ4037. Also, a second copy of that prophage was present in chromosome II of the pandemic strains and in strain AQ3810 (Figure 2B). In addition, a prophage region unique to the serotype O4:K68, pandemic strain AN5034 (AN5034_0425-0489) was identified by Phage_Finder (Table 2 and Additional file 1).

**Table 2:** Variable regions in *V. parahaemolyticus*

#	Region or insertion site relative to RIMD2210633	Number of ORFs	Function	RIMD (O3:K6 1996)	Peru466 (O3:K6 1996)	AN5034 (O4:K68 1998)	K5030 (O3:K6 2005)	AQ3810(O3:K6 1983)	AQ4037(O3:K6 1985)
**1**	Between VP0001-0002 (K5030_3039-3061)	23	DNA sulfur modification proteins	-	-	-	+	-	-

**2**	VP0197-0238	42	O3:K6 LPS/CPS	+	+	-	+	+	+

**3**	Replaced VP0197-0238 (AN5034_1849-1901)	53	O4:K68 LPS/CPS	-	-	+	-	-	-

**4**	Between VP0248-0249 (AN5034_1830-1837)	8	Unknown	-	-	+	-	-	-

**5**	VP0380-0403	24	Type I restriction endonuclease in tRNA-Met-1	+	+	+	+	-	-

**6**	VP0637-0643	7	Integrated element target tmRNA	+	+	+	+	-	-

**7**	Between VP0643-0644 (AN5034_1437-1442)	6	Integrated element target tmRNA	-	+	+	+	+	+

**8**	VP1071-1076	6	Unknown, contains phage integrase	+	+	+	+	-	-

**9**	VP1077-1087	11	Unknown, contains phage integrase	+	+	+	+	+	-

**10**	VP1385-1421	37	Type VI secretion system	+	+	+	+	-	+

**11**	VP1549-1562	14	Phage f237	+	+	+	+	-	-

**12**	Replaced VP1549-1562 (AQ4037_2432-2444)	13	Phage similar to f237	-	-	-	-	-	+

**13**	VP1563-1586	24	Phage alpha*	+	+	+	+	+	-

**14**	Between VP1604-1605 (AN5034_0425-0489)	65	Phage	-	-	+	-	-	-

**15**	VP1787-1865	78	Super integron	+	+	+	+	v	v

**16**	Between VP1864-1865 (K5030 1808-1814)	7	Addition to super integron, next to integrase	-	-	-	+	-	-

**17**	VP1884-1891	8	Unknown	+	+	+	+	-	-

**18**	VP1969-1974	6	Fatty acid and amino acid metabolism	+	+	+	+	-	+

**19**	VP2131-2144	14	Hypothetical proteins	+	+	+	+	-	-

**20**	Between VP2275-2276 (AQ4037_1749-1829)	81	*trh *pathogenicity island	-	-	-	-	-	+

**21**	Between VP2638-2639 (AQ4037_1361_1383)	23	Hypothetical proteins, contains integrase	-	-	-	-	-	+

**22**	VP2900-2910	11	Hypothetical proteins	+	+	+	+	-	-

**23**	VPA0434-0440	7	Hypothetical proteins	+	+	+	+	-	-

**24**	VPA0889-0912	24	Phage beta*	+	+	+	+	+	-

**25**	VPA1254-1270	17	Unknown	+	+	+	+	-	-

**26**	VPA1310-1398	86	*tdh *pathogenicity island	+	+	+	+	+	-

**27**	Replaced VPA1310-1313 (AN5034_A0845-A0851)	7	Hypothetical proteins	-	+	+	+	-	-

**28**	Replaced VPA1310-1398 (AQ4037_A1228-A1253)	25	Nutrient uptake and metabolism	-	-	-	-	+	+

In the first column, if genes are absent from RIMD, the gene numbers in one of the other genomes are indicated in the parenthesis.

* These two phages are very similar.

v Variable contents in the super integron

Each of the strains we studied had one or two integrated elements targeting the tmRNA gene (Table 2). For example, Peru466, AN5034, and K5030 had two different, integrated elements inserted in tandem into the 3' end of their tmRNA genes. The element closest to the tmRNA gene contained two genes that may influence virulence: a putative cyclic diguanylate phosphodiesterase EAL domain protein and an AraC superfamily putative fimbrial transcriptional activator. The second element was distinguished by the presence of a ribonuclease H-encoding gene. The first element was present in strains AQ3810 and AQ4037, but not in strain RIMD2210633. However, strains AQ3810 and AQ4037 lacked the ribonuclease H-encoding element present in strain RIMD2210633.

### Characterization of the pathogenicity islands

**Figure 3:**
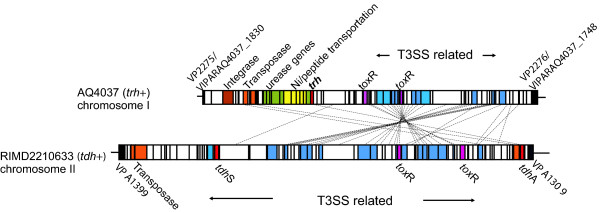
**Gene clusters in the *trh *and *tdh *pathogenicity islands**. The upper line indicates chromosome I of strain AQ4037, while the lower line indicates chromosome II of strain RIMD2210633. Thin lines connect homologous genes, and the boxes indicate ORFs. The colors denote various functional categories: brown, integrase; orange, transposase; green, urease-encoding gene; yellow, nickel/peptide transport-encoding gene; red, *tdh *and *trh*; pink, *toxR*; blue, T3SS-related gene; open box, other genes.

Pre-pandemic strain AQ4037 is *tdh*-, *trh*+ and urease-positive, and its genome sequence revealed a pathogenicity island (hereafter called *trh*PAI) containing 81 ORFs (Figure 3). Another pathogenicity island (hereafter referred to as *tdh*PAI) was previously identified in chromosome II of pandemic strain RIMD2210633 and includes loci VPA1310-1398 (Figure 3). *tdh*PAI contains a type III secretion system (T3SSII) and two copies of *tdh*; whereas, *trh*PAI contains *trh*, an integrase, transposases, a urease gene cluster, a peptide/nickel transportation system, and a T3SS that is different from the one in *tdh*PAI (Figure 3). The T3SS in AQ4037's *trh*PAI is similar to T3SSIIβ in *V. parahaemolyticus *TH3996, which is related to the T3SS in non-O1, non-O139 strains of *V. cholerae *[13]. Interestingly, *trh*PAI was found in chromosome II of strain TH3996, but it was located in chromosome I of strain AQ4037. This discrepancy in chromosomal location may be providing a clue to the pathogenicity island's mobility.

**Figure 4:**
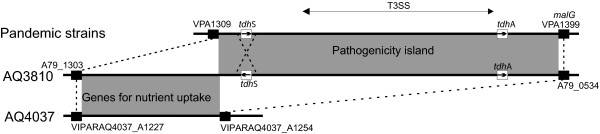
**Diagram of the *tdh *pathogenicity island**. Solid black boxes indicate the common border genes, and the homologous genes are connected by dotted lines. Homologous regions are shaded and *tdh *genes are indicated with arrows showing the direction of transcription. Other ORFs are omitted for simplicity.

Close examination of the *tdh*PAI region in the six genomes revealed major differences between the pre-pandemic and pandemic strains (Figure 4). The four epidemic strains' *tdh*PAIs were very similar to one another; however, the entire pathogenicity island was absent from the pre-pandemic, *tdh*- strain AQ4037. Instead, that strain contained a pre-pandemic-specific region of 18 ORFs important for the uptake and metabolism of carbon sources and other nutrients. In addition, although pre-pandemic strain AQ3810 contained both *tdh*PAI and the pre-pandemic nutrient uptake region, it had an inverted *tdh*S gene (Figure 4). Thus, the pre-pandemic and pandemic strains exhibited major differences between the region upstream of the pathogenicity island and in *tdh*'s orientation. Whether those variations affect the expression of the pathogenicity island's genes, which contributes to differences between the pre-pandemic and pandemic strains' virulence, remains to be determined. The pathogenicity island's organization suggests that an ancestral strain possessing the O3:K6 serotype may have recruited a *tdh*PAI next to VPA1309, which yielded a transient strain that subsequently lost the pre-pandemic island and gave rise to the pandemic strains. Another possibility is that the *tdh*+, pre-pandemic strain and the pandemic strains independently recruited the pathogenicity islands into the same location.

The *tdh *and *trh *genes are related but vary substantially [14], and many variants of *tdh *and two *trh *genes have been described. They have been found in various *Vibrio *species and their phylogenetic relationships are not in accordance with the relationship of the host species [14]. Although most of them are in the chromosomes, some of them are present in plasmids, a finding that is consistent with their proposed mobility. Also, the presence of *trh *and *tdh *on both chromosomes of *V. parahaemolyticus *supports the idea that they may have been acquired by lateral gene transfer and may have integrated into the bacterium's genome during independent events. Also, since *V. parahaemolyticus *strains that have both *tdh *and *trh *have been described [15], it might be worthwhile to sequence those strains' genomes in order to understand the evolutionary history of *tdh *and *trh *in their hosts.

TDH and TRH are the only confirmed virulence factors of *V. parahaemolyticus*; however, their precise roles are not well understood. Genomic sequencing revealed that the genes encoding those toxins are in close proximity to a T3SSII system, which suggests they may have the same origin as that transport system. Thus, it is tempting to speculate that TDH (and/or TRH) and T3SSII have coordinating activities related to the virulence of *V. parahaemolyticus*. Some translocon proteins and effectors have been identified for T3SSII [16,17]; however, the putative relationship between TDH, TRH, and T3SSII needs further investigation. Another T3SS (T3SSI) identified in the *V. parahaemolyticus *genome was demonstrated [8,18] to be required for cytolytic activity. However, T3SSI was conserved in all of the genomes we examined.

### Variability within the O3:K6 genetic locus

Since 1996, serotype O3:K6 has predominated among clinical isolates of *V. parahaemolyticus*, thus that serotype has been associated with the bacterium's increased virulence. However, strains of O3:K6 serotype had been isolated more than a decade before the pandemic initiated. It is not clear if there are variations within the O3:K6 genetic determinants between non-pandemic and pandemic strains, and thus causing subtle structural difference of the O and K antigens that could not be detected by the serotyping techniques. Therefore, we examined the O- and K-antigen-encoding gene clusters in the pre-pandemic and pandemic O3:K6 strains for any variations that may explain the observed increase in virulence. The O- and K-antigen-encoding gene clusters are juxtaposed in *V. parahaemolyticus *O3:K6 [19]. In strain RIMD2210633, they are located at loci VP0190-0238 in chromosome I (position 201,797-253,279) [20,21]. However, that region was conserved in all O3:K6 strains (sharing > 99.5% amino acid-encoding identity), which suggests that the serotype of *V. parahaemolyticus *may not be directly related to the pandemic strain's increased virulence.

### Serotype conversion from O3:K6 to O4:K68

**Figure 5:**
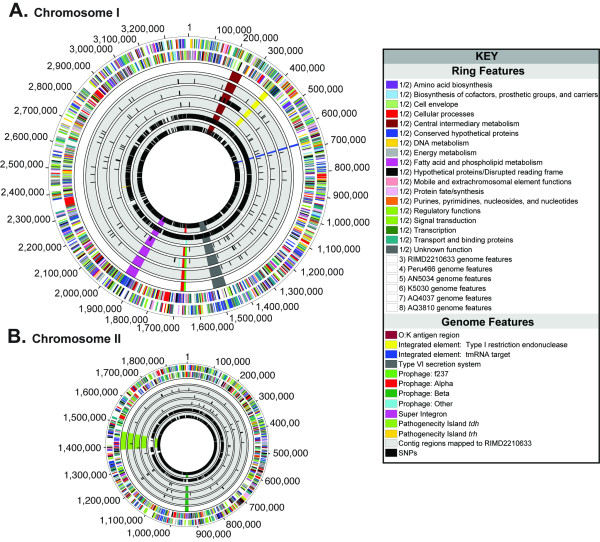
**Circular Illustration of Single Nucleotide Polymorphisms and Genome Features Relative to the Reference Strain RIMD2210633**. Chromosomes I (A) and II (B) are illustrated as a circles where each concentric circle represents genomic data and is numbered from the outermost to the innermost circle. Circles 1 and 2 represent RIMD2210633 ORFs and are colored based on function role categories. Circle 3 depicts the location in RIMD2210633 of various genomic features described in this study. Circles 4-8 denote the location of SNPs relative to RIMD2210633 and genomic features for strain Peru466, AN5034, K5030, AQ4037, and AQ3810, respectively. Refer to the key for details on color representations and circle numbers.

In addition to serotype O3:K6, > 20 other serotypes of *V. parahaemolyticus *were detected among pandemic strains [2]. The mechanism for this serotype conversion remains unknown. Since the O- and K-antigen loci are tightly linked on the chromosome [19], a recombination event involving this region would enable rapid conversion of serotypes. Before comparing the O- and K-antigen region between O3:K6 and O4:K68 serotypes, we first wanted to determine the variability of this region within the O4:K68 serotype as we did above for the O3:K6 serotype. We compared the O- and K- region of strain AN5034 (O4:K68), AN5034_1842-1901, to the O- and K- region of another O4:K68 strain designated NIID242-200 [21]. Both clusters were nearly identical (9 mismatches in a 63-kb-long region), as observed above for the O3:K6 loci. When comparing the O- and K-antigen regions between strain AN5034 (O4:K68) and strain RIMD2210633 (O3:K6), this region varied substantially, except for the first seven genes, suggesting recombination as a method for serotype conversion. To help identify the scope of the recombination event, we analyzed the distribution of single-nucleotide polymorphisms (SNPs) in the genome of strain AN5034 compared to strain RIMD2210633. Compared to the other pandemic isolates, strain AN5034 had 2,281 SNPs (excluding insertions and deletions), 2,142 (94%) of which clustered in a 141 kb region (position 199,786-341,273 in RIMD2210633 chromosome I, and position 166,252-324,726 in AN5034 contig ACFO01000016.1), corresponding to between 2-kb upstream and 88-kb downstream of the O- and K-antigen-encoding gene clusters (Additional file 2 blue highlight, Figure 5 circle 5). This observation suggests that a recombination event involving a much larger region than the O-antigen- and K-antigen loci occurred and gave rise to the new O4:K68 serotype during the pandemic.

### Origin of the South American outbreaks

Our results indicated that the pandemic strains are closely related to one another. Strain RIMD2210633 differed from strain K5030 and the Peruvian strain (Peru466) by 70 and 76 SNPs, respectively (Additional file 2). Peru466 was isolated at approximately the same time the first outbreak (which later evolved into a pandemic) was reported in India. Also, its gene content is almost identical to that of a pandemic Asian strain (RIMD2210633) isolated at the same time, which supports the idea that the pandemic strain spread from Asia to South America very soon after it emerged. Considering the proximity, it is likely that the 2005 outbreak in Chile was caused by strains descended from the Peruvian isolates. We speculate that the strains from South American outbreaks are closely related to the strains from Indian outbreaks. This hypothesis will require genomic sequencing of additional strains from South America, specifically those from Chile, to confirm.

Fewer cases of disease caused by *V. parahaemolyticus *were reported from Asia during the later stages of the pandemic than during its early stages (Nair, personal observation); thus, we suspect that the strains isolated during the later stages were not as virulent as those obtained during its early stages. Therefore, we sequenced and analyzed the genome of strain K5030, which was isolated from India almost a decade after the pandemic started, to look for gene deletions and variations. Major gene deletions were not identified in strain K5030; instead, several insertions were detected (Table 2). In addition to the cassettes added to the strain's SI, K5030 had an insertion of 23 ORFs (between VP0001 and VP0002) containing a DNA phosphorothioation (dnd) system, which incorporates sulphur into the DNA backbone, [22,23]. Recent evidence supports the lateral transfer of dnd genes among bacterial genomes [24]. Besides the dnd genes, there were also two transposases and one integrase in the insertion. However, it is not clear whether the addition of those ORFs reduced the strain's virulence by altering the structure and function of virulence factors.

### Comparative genomic analyses of the three major pathogenic *Vibrio *species

In order to characterize important genetic differences between *V. parahaemolyticus*, *V. cholerae *and *V. vulnificus*, we compared their core genomes. The *V. cholerae *group consisted of four completely sequenced genomes of toxigenic, serotype O1 strains: N16961, M66-2, MJ-1236 and O395 [25-27]. The *V. vulnificus *group contained two completely sequenced genomes of strains CMCP6 and YJ016 [28,29], and the *V. parahaemolyticus *group consisted of strain RIMD2210633 and the two pre-pandemic isolates (strains AQ3810 and AQ4037). The orthologs from each group were extracted by comparing all members of the group, after which the core genes from each group were compared to each other.

**Figure 6:**
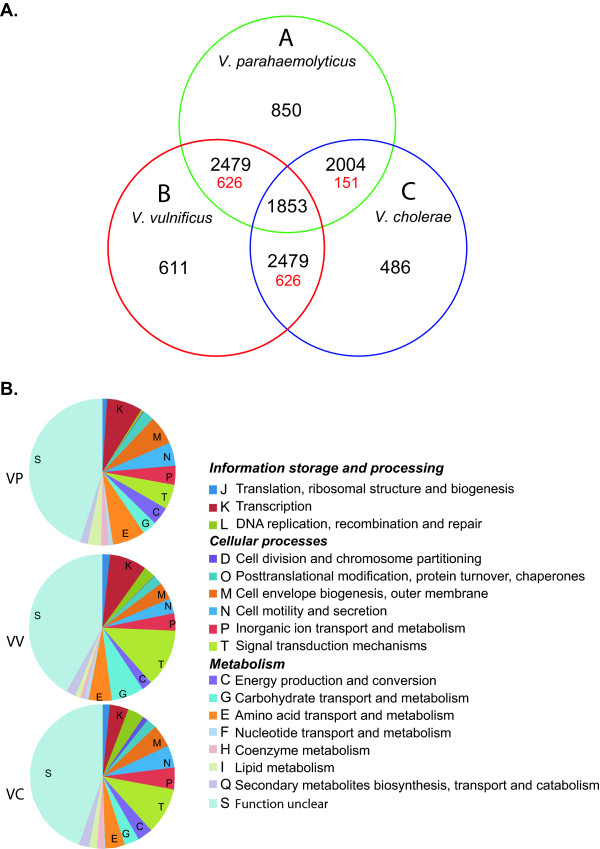
**Species-specific core genes of *V. parahaemolyticus*, *V. cholerae*, and *V. vulnificus***. Left panel: Each circle represents core genes extracted from selected strains of each species. The Venn diagrams show the number of shared proteins (black) and unique proteins (red) within a particular relationship for all three species. Right panel: Pie chart of species-specific genes. Functional categories are indicated with various colors and letters.

Forty-nine to 59% of the core genes in each species were common to all three species (Figure 6). However, 14-24% of the core genes in each group were only conserved in its own group and were in different COG (Clusters of Orthologous Genes) categories (Figure 6). These are likely to be the genes that define the bacteria at the species level. Furthermore, each species had specific genes and transporters required for various metabolic pathways, which indicates that they have different requirements for transporting various ions, nutrients, and other metabolites across their outer membranes. In addition, each species had unique two-component regulatory systems and chemotaxis genes, which indicate that they have specific signal pathways that respond to various environmental stimuli.

The three species also differ in their outer membrane structure and virulence genes (e.g. they had different sets of genes for surface polysaccharide biosynthesis). Furthermore, *V. vulnificus *has a unique set of genes for FLP pilus synthesis, and toxigenic strains of *V. cholerae *have genes for toxin co-regulated pilus synthesis, a well-studied virulence factor [27] (the *ctxØ *phage is not included as containing genes specific for toxigenic *V. cholerae *because it is not present in strain M66-2). *V. parahaemolyticus *possesses genes encoding two unique flagella, in addition to the genes required for the biosynthesis of flagella possessed by all three species. *V. cholerae *also has two different T3SS-containing islands [7]. T3SS has been reported to be closely related to T3SSII in *V. parahaemolyticus *and to be present in non-O1, non-O139 strains of *V. cholerae *[30,31], but we did not detect it in the toxigenic strains of *V. cholerae*. However, the possession of T3SS by *V. parahaemolyticus *and non-O1, non-O139 strains of *V. cholerae *suggests that this transport system is required for colonization of their unknown environmental hosts and reservoirs.

### Evolution of *V. parahaemolyticus*

In order to advance our understanding of the relationships between the *V. parahaemolyticus *strains we characterized, a set of 924 single-copy genes present in all six strains (plus an outlier of *V. vulnificus *CMCP6), taken from the analysis of three pathogenic Vibrios, was compiled, and a nucleotide maximum-likelihood tree was inferred for the concatenated set of 924 genes (Additional file 3). Our expectation that a resolved phylogeny supported by the majority of genes would be found and interpreted as an estimate of the strains' phylogenies was not met, a result similar to that of Boyd et al 2008 [9] who also found that all of the pandemic strains were intermixed with pre-pandemic strains, but used just three genes in their analysis. Inspection of the aligned genomes with the SNPs marked in each one (Figure 5) revealed that the SNPs were often clustered, possibly indicative of recombination events. Recombination events involving multiple SNPs would distort the branch lengths and provide a poor estimate of the phylogeny. In retrospect, this result might have been expected because we have shown that recombination events can involve a large number of variable nucleotides in numerous genes and each one of those events will distort the distance measure used for phylogenetic trees that assume all mutations are independently acquired. In the sequenced strains, the single recombination event that converted the serotype and replaced the neighboring 90 kb contained 14 times the total number of variable nucleotides observed in the rest of the genome (Additional file 2, Figure 5 circle 5). Thus, if only one of the 110 genes from this region were included with the rest of the genes in the genome to calculate a tree, that one gene, on average, would contribute 11% of the total variation. In order to calculate accurate trees, each recombination event and independent mutation event must be given equal weight.

## Conclusion

This study helps to improve our understanding of how *V. parahaemolyticus *evolved during a pandemic. The results of our multiple genome analyses are consistent with the idea that pandemic strains of *V. parahaemolyticus *evolved from pre-pandemic strains by numerous deletions and acquisitions of genetic material. Pandemic strains differ from pre-pandemic strains mostly in mobile genetic elements and the structure of the pathogenicity islands. Serotype conversion to O4:K68 was likely due to a recombination event involving a region much larger than the O-antigen- and K-antigen-encoding gene clusters. In addition, this study revealed that (*tdh*+ *trh*-) and (*tdh*- *trh*+) strains not only have different toxin genes, but also differ in the structures and locations of their pathogenicity islands.

Lateral gene transfer seems to be the major force shaping the virulence of *V. parahaemolyticus*, as evidenced by the diversity in the locations and nucleotide sequences of the virulence factor-encoding genes. In addition, previous studies had shown that insertion sequences in *V. parahaemolyticus *could change the genome structure and result in the loss of a major virulence factor [32,33]. However, the pandemic strains we studied are almost monomorphic (except for pathogenicity islands and mobile elements) and the outbreaks in Asia and South America are closely related. During the pandemic's later development, pandemic strains with the O3:K6 serotype (and its serovariants) were no longer prevalent in India, where the pandemic originated (Nair, personal observations). Instead, massive outbreaks were reported in Chile. This observation suggests that the virulent clone that spread to South America during the pandemic's early stage has persisted in that area and continues to cause outbreaks. There was no loss of known virulence genes in the later stage pandemic (K5030) isolate from south Asia; therefore, its virulence status needs further evaluation.

Genetic changes in the etiologic agent may not be the only factor leading to *V. parahaemolyticus*-mediated pandemics (e.g. optimal environmental conditions may enable pandemic strains to flourish in their reservoirs). For example, an outbreak of foodborne diarrheal disease caused by *V. parahaemolyticus *was reported on an Alaskan cruise ship during 2004, and the source of the infection was *V. parahaemolyticus*-contaminated oysters harvested following warm weather in Alaska, where *V. parahaemolyticus *had not been previously isolated [34]. In addition, although there are major genetic differences between pre-pandemic and pandemic strains of *V. parahaemolyticus*, and they may, to some extent, contribute to the pandemic strains' increased virulence, a pathogenicity island (which contains *tdh *and T3SS) is also present in the pre-pandemic strain AQ3810. Thus, it is likely that the recent *V. parahaemolyticus*-mediated pandemic resulted from the convergence of genetic changes in the etiologic agent and the presence of optimal conditions for survival and growth in its natural reservoirs.

## Materials and methods

### Strain isolation and verification

The *V. parahaemolyticus *strains were isolated on TCBS (thiosulfate, citrate, bile salts, and sucrose) agar medium followed by their presumptive identification with a multitest medium [35]. The strains' identities were confirmed by a species-specific *toxR *assay [29], a commercially available *V. parahaemolyticus *antiserum kit (Toshiba Kagaku Kogyo Co., Ltd., Tokyo, Japan) was employed for serological typing, and *tdh *and *trh *were identified by PCR [36]. Strains were cultured overnight in Luria-Bertani broth, and DNA was obtained by lysing the bacteria with proteinase K followed by DNA extraction and purification with a Qiagen Maxi Kit (Valencia, CA).

### Genome sequencing

The genomes of *V. parahaemolyticus *strains AN5034, AQ4037, K5030, and Peru466 were sequenced by the Sanger whole-genome random shotgun method [37]. Briefly, one small insert plasmid library (3-4 kb) and one medium insert plasmid library (10-12 kb) were constructed by random nebulization and cloning of genomic DNA. During the initial random-sequencing phase, 8-fold sequence coverage was achieved with the small- and medium-size libraries sequenced to yield 5-fold and 3-fold coverage, respectively. The sequences were assembled using the Celera Assembler [38], and ordered scaffolds were generated by using NUCMER [39] to align the contigs to the genome of *V. parahaemolyticus *RIMD2210633, followed by hierarchical scaffolding with BAMBUS [40]. The contig N50 was determined as described [41].

An initial set of open-reading frames (ORFs) was identified using GLIMMER [42], and ORFs shorter than 90 bp (and some with overlaps) were eliminated. A region containing the likely origin of replication was identified, and base pair 1 was designated adjacent to the *dnaA *gene located in that region [43]. The ORFs were searched against a nonredundant protein database, and the ORF predictions and gene family identifications were done as previously described [27]. Two sets of hidden Markov models (HMMs) were used to determine ORFs membership in families and super families. These included 721 HMMs from Pfam v22.0 and 631 HMMs from the TIGR ortholog resource. A transmembrane hidden Markov model (TMHMM) [44] was used to identify membrane-spanning domains in proteins, and putative functional role categories were assigned internally as previously described [45].

The nucleotide sequences and the corresponding automated annotations for the genomes of *V. parahaemolyticus *strains AN5034, AQ4037, K5030, and Peru466 were submitted to NCBI, with accession numbers [GenBank:ACFO00000000, Genbank:ACFN00000000, Genbank:ACKB00000000, and Genbank:ACFM00000000], respectively.

### Comparative genomics

The database and cut-offs mentioned above were used, as previously described [37], to (i) produce an ortholog match table, (ii) construct a Venn diagram, and (iii) bin the relationships within the Venn diagram. Synteny plots using PROMER [39] were computed as previously described [37]. SNPs were identified by mapping chromosomal contigs to the complete reference genome of RIMD2210633 using NUCMER [39] with default setting and displayed using the SHOW-SNPS tool with the -C (do not report SNPs from alignments with an ambiguous mapping) and -I (do not report indels) options. SHOW-SNPS is part of the MUMMER 3 distribution http://mummer.sourceforge.net/. DNA maximum likelihood trees were created (using PAUP* 4.0b) for each of the 924 entries in the above mentioned ortholog table that had orthologs for *V. vulnificus *CMCP6 and the six *V. parahaemolyticus *strains. In order to ensure proper alignment of the coding regions, the trees were based on DNA alignments back-aligned from the proteins' alignments.

## Competing interests

The authors declare that they have no competing interests.

## Authors' contributions

OCS, RH, GBN, AIG, MN, and DEF conceived and organized the project; GBN, AIG, and MN provided strains; OCS and YC isolated DNA; DEF organized the sequencing studies; DEF, JHB, and YC analyzed the genomic data; and YC, DEF, JHB, and OCS prepared the manuscript. All authors read and approved the final manuscript.

## Supplementary Material

Additional file 1 ***Vibrio parahaemolyticus *Ortholog Match Table**. BLAST-based ortholog Match Table of *V. parahaemolyticus *strains Click here for file

Additional file 2 ***Vibrio parahaemolyticus *Single Nucleotide Polymorphisms**. Single Nucleotide Polymorphisms of *V. parahaemolyticus *genomes relative to reference strain RIMD2210633 Click here for file

Additional file 3 **Relationships of *V. parahaemolyticus *strains**. DNA Maximum Likelihood tree based on 924 orthologs of the six *V. parahaemolyticus *strains. *V. vulnificus *strain CMCP6 was used as an outlier. Click here for file
